# Psychological Resilience in Individuals with Type 2 Diabetes Mellitus in Jordan Is Associated with Insomnia Severity: A Cross-Sectional Analysis

**DOI:** 10.3390/jcm15020880

**Published:** 2026-01-21

**Authors:** Omar Gammoh, Mervat M. Alsous, Hazem Gammoh, Alaa A. A. Aljabali, Hanan Abu Alshaik, Renad Alrtemat, Mariam Al-Ameri, Abdullah M. AlNsour, Rami Farraj, Amal Ireifij, Alaa A. Alsharif, Nada A. Alsaleh

**Affiliations:** 1 Department of Clinical Pharmacy and Pharmacy Practice, Faculty of Pharmacy, Yarmouk University, Irbid 21163, Jordan; renadrey899@gmail.com (R.A.);; 2 Department of Biopharmaceutics and Clinical Pharmacy, Faculty of Pharmacy, The University of Jordan, Amman 11942, Jordan; 3 Healthy Community Clinics Program, Royal Health Awareness Society, Amman 11942, Jordan; hgammoh@rhas.org.jo (H.G.);; 4 Department of Pharmaceutics and Pharmaceutical Technology, Faculty of Pharmacy, Irbid 21165, Jordan; alaaj@yu.edu.jo; 5 Prince Hamza Hospital, Amman 11947, Jordan; 6 Department of Pharmacy Practice, College of Pharmacy, Princess Nourah bint Abdulrahman University, Riyadh 11671, Saudi Arabia

**Keywords:** psychological resilience, type 2 diabetes mellitus, insomnia, mental health

## Abstract

**Background/Objectives**: Although psychological resilience is an important factor related to mental health outcomes, it is understudied in patients with long-term health conditions in developing countries such as Jordan. Type 2 diabetes mellitus (T2DM) is rapidly increasing in Jordan; thus, its prevalence and correlation with psychological resilience are worth investigating. Through this study, we aimed to estimate the prevalence of psychological resilience levels and identify their associated factors in people with T2DM in Jordan based on demographic data, clinical data, and insomnia severity. **Methods**: Participants were sampled from the patient register of the Royal Health Awareness Society (RHAS), which permitted access to people receiving T2DM-related services. **Results**: Data from 350 individuals with T2DM were analyzed; 185 (52.9%) were female, 276 (78.9%) were over 50 years of age, and 308 (88%) were married. In total, 56 (16%) people reported moderate–severe insomnia, and 64 (18.3%) reported low resilience. Being more resilient was found to correlate with “being married” (OR = 3.33, 95% CI (1.60–6.89), *p* = 0.001) but was negatively correlated with “moderate–severe insomnia” (OR = 0.21, 95% CI (0.11–0.40), *p* < 0.001). **Conclusions**: Addressing insomnia in individuals with T2DM is warranted to improve their resilience levels, thus promoting their mental health.

## 1. Introduction

The mental health of individuals diagnosed with type 2 diabetes mellitus (T2DM) is an emerging global issue. Resilience is defined as having the capacity to rebound from adversity, and it is considered a significant measure of psychosocial processes and quality of life of people with chronic illness, such as hypertension, cancer, heart failure [1], and T2DM [2]. The existing evidence relates resilience to a set of biological alterations that are associated with behavioral disruptions, especially in the presence of long-term medical conditions [3,4,5].

Better resilience has been related to better mental health status, such as lower levels of depression or perceived stress [6,7], improved quality of life, and decreased use and cost of medical management [8]; however, there is limited evidence suggesting that resilience and physical health outcomes are related [9].

In Jordan, T2DM is recognized as a prevailing chronic health condition. Based on the Jordanian National Centre for Diabetes, Endocrinology, and Genetics, the combined prevalence of diabetes mellitus (DM) and pre-DM could be estimated to affect 45% of adults [10]. Findings from the 2019 Jordan STEPwise Survey further highlight this burden: 20.5% (95% CI: 17.2–23.8) of adults aged 45–69 years had raised fasting blood glucose or were on medication, while 14% of all adults tested showed above-normal glucose levels, including 6.1% with impaired fasting glucose (pre-DM), which represented nearly 43% of those with abnormal values (Jordan Ministry of Health & WHO, 2020 [11]). Regionally, the Middle East and North Africa (MENA) bear the highest T2DM prevalence worldwide, with 16.2% of adults—equivalent to 73 million people—living with T2DM in 2021. Globally, the burden is equally alarming: the prevalence of T2DM among adults was estimated at 9.3% (463 million people) in 2019, and it is projected to rise to 578 million by 2030 and 700 million by 2045 [12]. The aforementioned health statistics are further affected by the economic burden [1]; over recent decades, the frequency of people with T2DM has increased, from approximately 108 million in 1980 to more than 422 million in 2014, driven largely by aging populations and rapid demographic growth [13].

People with T2DM could experience low quality of life. Apart from complex self-management and therapeutics, T2DM also contributes considerably to the lifestyle of a patient [2]. Difficulty in regulating blood glucose levels in people with T2DM has the potential to cause significant problems, such as neuropathy, heart disease, nephropathy, and other problems [14]; therefore, in order to efficiently manage T2DM, prevent long-term effects, and boost quality of life, people with T2DM should be cared for appropriately [15].

Therefore, the proper management of diabetes enhances resilience, which, in turn, helps to lower diabetes distress [16]. Stress is a significant psychosocial aspect that may harm people’s health [17]. Previous studies have shown that T2DM distress, a comprehensive and multidimensional term that includes emotional burden, interpersonal distress related to the illness, regimen-related distress, and physician-related distress, can affect resilience in people with T2DM [16]. Previous research has shown that high levels of distress affect 44.6% of people with T2DM worldwide [18]. Previous research has demonstrated that, when distress progresses, resilience declines [19,20].

Insomnia, a well-known sleep disorder, is characterized by difficulty in falling asleep or difficulty in maintaining quality sleep, and early awakenings. Insomnia is closely related to medical conditions such as T2DM [21].

Insomnia and resilience have been extensively studied in the last few decades on different biological and environmental levels [22]. Recent evidence suggests that poor sleep quality is one of the mechanisms underlying poor psychological resilience, underscoring the importance of the neuroendocrine response system to stress [23].

Since T2DM is a national challenge in a developing country such as Jordan, the Royal Health Awareness Society (RHAS), in collaboration with the Jordanian Ministry of Health, is conducting The Healthy Community Clinic (HCC) Project, which is a national initiative designed to strengthen the prevention and management of non-communicable diseases (NCDs). The HCC program provides access to people with T2DM.

Although numerous studies have been conducted on T2DM in Jordan, to the best of our knowledge, no previous study has attempted to explore the prevalence and identify the determinants of resilience in individuals diagnosed with T2DM in Jordan. The authors hypothesized that demographic variables and insomnia could be associated with resilience levels in this population; based on the outcomes of this study, future steps can be adopted to address the factors related to resilience, thus ensuring optimal care plans.

Therefore, the current cross-sectional investigation aimed to answer the following three questions about psychological resilience in a cohort of people with T2DM: (1) What is the estimated prevalence of poor psychological resilience? (2) What demographic and clinical factors are associated with psychological resilience levels? (3) Is insomnia associated with poor resilience?

## 2. Materials and Methods

**Study design and settings**

For this cross-sectional investigation, we recruited people with T2DM from the RHAS database through the random sampling technique as part of a large mental health research project on T2DM. The RHAS database is used to compile records of individuals enrolled in RHAS-affiliated T2DM and primary health clinics across multiple Jordanian governorates that are part of the Healthy Community Clinics (HCCs); it includes demographic details, clinical information related to T2DM, and contact information of people who consented to be followed up as part of the Royal Health Awareness Society (RHAS) national health awareness program. The database is maintained in collaboration with the Ministry of Health under RHAS data governance policies. The research team members contacted the participants via phone calls, and willing participants were guided through all the sections of the study questionnaire. The data collection period was between June and July 2025. To calculate the sample size needed, G-power software was used based on logistic regression, a confidence level of 0.95, and a confidence interval of 0.05. The G-power revealed that 238 participants were required as a minimum, calculated according to [24]; therefore, the researchers aimed to recruit a higher number of participants.

**Inclusion criteria:**

People with T2DM registered on the RHAS database, who were aged above 18 years old, currently residing in Jordan, and who fully completed the study instrument were included.

**Exclusion criteria:**

People with other types of diabetes, such as type 1 or gestational diabetes, were excluded from the study; additionally, people who did not complete all the sections of the study were excluded.

**Ethical Considerations**

The study protocol was approved by the Ministry of Health (number 5889) and the Yarmouk University IRB (number 479/2024). The Royal Health Awareness Society (RHAS) granted permission to use its patient database for recruitment and data collection purposes. All participants signed an electronic consent form before completing the study. All data were kept anonymous, and all participants possessed the right to retract at any time.

**Study instrument**

The study instrument included the following different sections: (1) consent form, (2) demographic data, (3) T2DM-related clinical information, (4) insomnia, and (5) resilience (outcome variable). The consent form outlined the study objectives, sections, and estimated time for completion of the study.

**Covariates**

The demographic information section consisted of structured questions about the participants’ sex, age, marital status, educational achievement, residence, and employment status. The clinical section consisted of questions exploring the duration of diagnosis with T2DM (less than five years, or five years or more), and participants had to report any comorbidities, including hypertension and dyslipidemia; in addition, questions were asked regarding adherence to regular HBA1C testing, the current status of HBA1C, and whether it was controlled (<7%) or uncontrolled (≥7%), as well as reasons for not controlling glycated hemoglobin (HBA1C) levels, with included answers such as “I cannot adapt to lifestyle changes for diet, exercise, and medication adherence” and “other reasons.” Moreover, questions about the services offered were asked as follows: “Are you aware of the clinic services for T2DM people?” Responses were “yes,” perhaps,” or “no.” “Do you think that the follow-up calls from the clinics are effective regarding your health?” Answers included “yes” and “perhaps.” Finally, the clinical section explored the T2DM medications received, classified as follows: “metformin alone”, “metformin and sulfonylurea”, and “insulin”. The demographic and clinical questions were based on previous similar studies [25,26].

Insomnia was another covariate; it was assessed using the Insomnia Severity Index—Arabic version (ISI-A); this reliable scale (Cronbach’s alpha 0.84) captures the different symptoms of insomnia over the last two weeks. The scale comprises seven questions, with Likert Scale answers ranging from “never” to “all the time”, with a numerical score corresponding to each answer, thus generating a score range from 0 to 28, with a higher score indicating higher severity. The scores are interpreted as follows: “0–7”, no clinically significant insomnia; “8–14”, subthreshold insomnia; “15–21”, moderate severity; and “22–28”, severe insomnia. In this study, we grouped people scoring ≥15 to represent “moderate–severe insomnia” [27,28]. The Cronbach’s alpha for ISI-A in this study was 0.94.

**Outcome variable**

The outcome variable, resilience, was examined using the Connor–Davidson Resilience Scale (CD RISC), originally developed by Connor and Davidson [29]; the scale has been translated into Arabic [30] and validated among the Jordanian population with a content validity index of 0.88 [31]. The tool focuses on positivity, adaptation capabilities, and persistence through 25 structured queries, each rated against a Likert Scale answer with a score from 0 to 100; it is accepted that a score ≥ 50 indicates higher resilience according to the existing literature [32,33]; therefore, to estimate the prevalence of higher resilience, the scale was treated as a categorical variable. Each resilience factor contributes differently to the overall resilience score, which is summed from different Likert Scale answers ranging between “never” and “always”. The Cronbach’s alpha for the CD-RISC in this study was 0.946.

**Statistical analysis**

The study sample is described by using frequencies and percentages for demographic data, clinical data, and insomnia severity across resilience levels, which were treated as categorical variables, as shown in Table 1. A preliminary univariate binary logistic regression analysis, shown in Table 2, was used to identify potentially confounding variables showing *p* < 0.10, which were included in a multivariable binary logistic regression model that was built using the backward stepwise model. The final model included only significant (*p* < 0.05) independent variables associated with higher resilience (the dependent variable), as shown in Table 3. Significance was set at *p* < 0.05 and confidence intervals at 95%. Data was analyzed using SPSS (Version 23 by IBM, Armonk, NY, USA).

## 3. Results

### 3.1. Description of the Study Sample

Analysis of the 350 participants with T2DM revealed that 185 (52.9%) were female, 276 (78.9%) were aged ≥ 50 years, 308 (88%) were married, 272 (77.7%) had received primary and high school education, 170 (48.6%) were unemployed, and 62 were employed (51.4%). The participants resided mainly in Amman (109 [31.1%]), Mafraq (128 [36.6%]), and Irbid (65 [18.6%]). Clinically, 220 (62.9%) participants were diagnosed with hypertension, 69 (19.7%) were diagnosed with dyslipidemia, and 229 (65.4%) had been diagnosed with T2DM for >5 years. In addition, 287 (82%) participants reported adhering to regular HbA1C routine checks, and 173 (51.2%) reported their HbA1C to be “controlled.” Additionally, 144 participants (41.1%) reported difficulties in implementing lifestyle changes regarding diet, exercise, and medication adherence. Moreover, 148 (42.3%) were unaware of the healthy community clinic services provided by the MoH and supported by RHAS, and 207 (59.1%) reported that follow-up phone calls were ineffective in T2DM management. The aforementioned findings directly implicate the delivery of services from the RHAS and define possible routes through which to strengthen communication, patient education, and the efficacy of calls. Relating the results back to the RHAS programs is relevant, considering the potential of these programs as agents to carry quality improvement plans into the future. Regarding T2DM medications, 148 (42.3%) participants were taking metformin, 166 (47.4%) were taking metformin with sulfonylurea, and 57 (16.3%) were taking insulin. There were significant differences in the medication use patterns. Of the insulin users, 68% admitted to occasionally skipping the drug, while 45% of the oral medicine users had the same habit. The barriers they reported included concerns about cost (32%), fear of side effects (28%), and complicated dosing schedules (24%). Finally, 56 (16%) participants reported moderate–severe insomnia according to the ISI-A, and 64 (18.3%) reported low resilience levels according to the Davidson–Connor Scale. Table 1 provides the frequencies and percentages across resilience levels.

### 3.2. Factors Associated with Resilience Levels

The factors associated with resilience levels were examined using preliminary univariate logistic regression analysis to identify potential confounders with *p* < 0.1; these were “female,” “married,” “moderate–severe insomnia,” and “follow-up phone call effectiveness,” as shown in Table 2.

The above independent factors were used to construct the multivariable regression model that finally revealed only two independent factors to be associated with resilience levels, as follows: higher resilience was significantly and positively associated with “married” people (OR = 3.32, 95% CI (1.60–6.89), *p* = 0.001), and significantly and negatively associated with “moderate–severe insomnia” (OR = 0.21, 95% CI (0.11–0.40), *p* < 0.001). The model showed Nagelkerke R^2^ = 0.14.

## 4. Discussion

In this study, we aimed to estimate the prevalence of psychological resilience levels and identify their likely associated factors in people with T2DM in Jordan based on demographic data, clinical data, and insomnia. According to the results, approximately 18% of the sample population exhibited low resilience. High resilience, on the contrary, was correlated with being “married” and negatively correlated with “moderate–severe insomnia.”

Through this investigation, we identified factors that are likely associated with the psychological resilience of people with T2DM; this is one of the very few studies examining the prevalence and determinants of resilience in Arab people with T2DM. Similar studies about resilience in Arab people with T2DM are still lacking; one descriptive study on people with T2DM described the psychological resilience level in these people as “moderate” resilience, with no investigation of the underlying risk factors [34]; therefore, comparing the findings of the current study with the previous and recent literature remains challenging.

Insomnia was a significant factor associated with poor resilience; people with moderate–severe insomnia were almost 80% less likely to be psychologically resilient than those without insomnia; the connection between insomnia and resilience, therefore, is established. In other words, according to the existing literature, people with insomnia usually report low resilience compared to people not reporting insomnia; for instance, one study recruiting 58 people with insomnia demonstrated that people diagnosed with insomnia reported poor resilience scores versus healthy subjects [35]. In addition, one recent large-scale follow-up trial comprising > 600 participants concluded that improving insomnia through a cognitive behavior therapy approach led to an improvement in the resilience of people diagnosed with insomnia [36]. An important dimension of this study is examining insomnia and psychological resilience among a cohort of people with T2DM. An important concern is sleeplessness amongst people with T2DM due to neuropathic pain, nocturia, and changes in glucose levels [37]. Adaptation to T2DM life challenges is hindered in people with poor sleep quality, which worsens mood disorders, fatigue, and glycemic control [38]. The results are consistent with earlier published studies, noting that sleep disturbances precede psychological vulnerability and correlate with T2DM, decreasing protection from stress and resilience [39,40,41]. Look AHEAD Trial results on psychological resilience in older people with T2DM established that improved mental quality of life and fewer depressive symptoms were associated with increased psychological resilience [42]. Another study directly emanates from their capacity to cope with pressure and stay positive, a necessary reference point in the enhancement of the quality of sleep in people with T2DM [39].

Apart from psychological aspects, the underlying relationship between insomnia and poor resilience could also be based on a complex and intriguing biological basis, of which inflammation could play a major role, especially in people with T2DM. Insomnia in people with T2DM was associated with increasing odds for worsening glycemic control as part of the metabolic syndrome experienced by these people, and, more interestingly, insomnia was linked to elevated levels of C-reactive protein, a general marker for inflammation [43]. Moreover, it has been demonstrated that insomnia is related to activating the nuclear factor (NF)-κB, which plays a critical role in inflammation [44]; resilience, on the other hand, serves to overcome stressful events. Extensive evidence exists on the tight bond between stress and immune factors secreted from the central nervous system and the immune system [45]. In this context, the immune–gastrointestinal microbiota mediated by the inflammatory players and their interactions is highly implicated in resilience [46].

Regression analysis indicates that resilience was positively correlated with marital status. Compared with single people, married people were more than three times more likely to exhibit higher resilience. Marriage can be a crucial source of joint accountability, emotional support, and motivation for adhering to treatment [47]. A spouse’s presence can reduce stress and feelings of loneliness, improve T2DM self-efficacy, and encourage more participation in healthy lifestyle choices [48]. One study concluded that support from family and a spouse or partner can help increase adherence to the medication and lifestyle changes needed to attain ideal glycemic control and prevent related problems. Therefore, healthcare systems should prioritize evidence-based methods concerning marital and social support to help individuals overcome adversity. The authors acknowledge that the quality of the marriage relationship was not assessed; therefore, this finding cannot be generalized.

At the service level, the presented outcomes highlight several possible avenues through which Healthy Community Clinics (HCCs) could reinforce comprehensive mental health care for people with T2DM, by implementing psychological care units that could offer awareness and counseling sessions, whether one-to-one or through focus groups, thereby strengthening psychological health, self-care behavior, and long-term outcomes in this group of people.

In addition, one interesting covariate investigated was the duration of T2DM diagnosis. In this study, 65% of the participants reported being diagnosed with T2DM for more than five years. Although T2DM duration was not significantly associated with resilience, this is worth future investigation because longer duration of disease could be related to either proper adaptation or maladaptation to a healthy lifestyle; also, T2DM duration could span from one year to decades, thus presenting an attractive component of resilience research and its impact on psychological status using follow-up studies [49].

Although the present cross-sectional investigation provides new insights, it has some limitations. For example, the study is based on data from self-report screeners, but it was not corroborated by a sleep study or a more in-depth clinical assessment by psychologists who can examine a sleep diary, the sleep environment, or any insomnia-inducing medications or daily habits; however, the quality of the data in the present study has been maintained through the use of validated scales, representable sample size, and robust statistical analysis. Moreover, the study followed a cross-sectional design, which prohibited future follow-up for the participants. However, cross-sectional studies are useful to identify issues that can be further studied in longitudinal studies. Another limitation was in the regression model, where the Nagelkerke R^2^ value was 0.14, i.e., it explained only 14% of the outcome; indeed, this means that several factors are yet unexamined in relation to resilience; perhaps depression, anxiety, stress, and other factors could be in the scope in the future.

Furthermore, the heterogeneity of resilience response among individuals is well recognized; the personal resilience outcome is a dynamic process and can be positive or negative, depending on many circumstances, such as personal experience towards adversity and the interaction between genes and environment [50]. Although a validated tool was used to screen resilience levels, the cut-off points provide only estimations to discriminate between high and low resilience, based on the research objective and the supporting literature; therefore, this was a limitation of the study, due to the potential for inaccuracy. We also acknowledge that even participants with low resilience levels, as defined in our study, could also report significant insomnia due to other factors, such as stress, anxiety, and depression, that were not examined in the current study; this also explains, in part, the modest explanatory power of the regression model (Nagelkerke R^2^ = 0.14).

Thus, future studies could use a longitudinal design to measure the changes in resilience; furthermore, the findings of this research provide insights for policymakers and active societies on the ground, such as the RHAS, to implement mental health-oriented services in their national strategies, thus raising awareness among healthcare providers and people with medical conditions, especially those with T2DM.

The findings of this study underline the importance of screening mental health in this fragile population and have significant implications; thus, it is necessary to develop innovative solutions to integrate psychological care, either virtually or via a face-to-face approach.

## 5. Conclusions

In this cross-sectional study, we provide an estimation of resilience levels and their most likely associated factors in a cohort of people with T2DM, considering their demographic and clinical characteristics and insomnia severity. The significant negative association between insomnia and resilience underscores the critical role of sleep quality in the management of chronic conditions. These findings suggest that sleep disturbances should be addressed as an integral part of T2DM care and patient support programs. Moreover, this screening preliminary cross-sectional study highlights the need to expand research on the broader spectrum of sleep disorders among people with non-communicable diseases, as these conditions may further impact resilience, adherence to treatment, and overall health outcomes. Future investigations should combine qualitative and quantitative approaches to identify modifiable factors, inform targeted interventions, and guide health policy and service delivery improvements for people with NCDs.

The policy implications extend beyond the treatment of specific people. Healthcare financing models should encourage resilience screening and intervention. Educating healthcare professionals on resilience assessment methods may reduce long-term costs while improving patient outcomes. The inclusion of mental health specialists in the T2DM care team represents a structural shift with proven advantages.

## Figures and Tables

**Table 1:** Distribution of covariates across the resilience levels of the study participants (n = 350).

Factor	Category	Lower Resilience n (%) 64 (18.3%)	Higher Resilience n (%) 286 (81.7%)
Sex	Male	22 (34.4)	143 (50)
	Female	42 (65.6)	143 (50)
Age	Below 50 years	16 (25)	58 (20.3)
	50 years and above	48 (75)	228 (79.7)
Marital Status	Single	16 (25)	26 (9.1)
	Married	48 (75)	260 (90.9)
Education	University level	13 (20.3)	65 (22.7)
	High school education	51 (79.7)	221 (77.3)
Governorate	Amman	21 (32.8)	88 (30.8)
	Irbid	15 (23.4)	50 (17.5)
	Salt	9 (14.1)	8 (2.8)
	Mafraq	14 (21.9)	114 (39.9)
	Ma’an	1 (1.6)	15 (5.2)
	Zarqa	4 (6.3)	11 (3.8)
Employment	Employed	11 (17.2)	51 (17.8)
	Unemployed	36 (56.3)	134 (46.9)
	Retired	17 (26.6)	101 (35.3)
Insomnia status	Mild and below	59 (63.4)	283 (86.3)
	Moderate–severe insomnia	34 (36.6)	45 (13.7)
Comorbid hypertension?	No	24 (37.5)	106 (37.1)
	Yes	40 (62.5)	180 (62.9)
Comorbid dyslipidemia?	No	51 (79.7)	230 (80.4)
	Yes	13 (20.3)	56 (19.6)
Diagnosed with T2DM since	Less than 5 years	25 (39.1)	96 (33.6)
	5 years and above	39 (60.9)	190 (66.4)
HbA1C status	Controlled	34 (56.7)	139 (50)
	Uncontrolled	26 (43.3)	139 (50)
Why unable to control HbA1C?	Hard to change lifestyle (diet, physical exercise, and medication)	21 (32.8)	123 (43.0)
	Other reasons	43 (67.2)	163 (57.0)
Are you aware of the clinic services for T2DM people?	No	25 (39.1)	123 (43.0)
	Perhaps	39 (60.9)	163 (57.0)
Do you think that the follow-up calls from the clinics are effective regarding your health?	No	30 (46.9)	177 (61.9)
	Perhaps	34 (53.1)	109 (38.1)
Using metformin	No	32 (50)	170 (59.4)
	Yes	32 (50)	116 (40.6)
Using metformin + sulfonylurea	No	37 (57.8)	147 (51.4)
	Yes	27 (42.2)	139 (48.6)
Using insulin	No	52 (81.3)	241 (84.3)
	Yes	12 (18.8)	45 (15.7)

**Table 2:** Univariate logistic regression analysis of covariates, with “higher resilience” as the dependent variable.

Covariate	uOR	95% CI	*p*-Value
Female	0.52	0.29–0.92	0.02 *
Age > 50 years	1.31	0.69–2.47	0.40
Married	3.33	1.66–6.67	0.001 *
Highschool education	0.86	0.44–1.69	0.67
Governorate	1.13	0.94–1.35	0.18
Unemployment	1.18	0.80–1.73	0.40
Moderate–severe insomnia status	0.21	0.11–0.39	<0.001 *
Comorbid hypertension	1.01	0.58–1.78	0.94
Comorbid dyslipidemia	0.95	0.48–1.87	0.89
Diagnosed with T2DM for ≥5 years	1.27	0.72–2.22	0.40
Uncontrolled HbA1C status	1.30	0.74–2.29	0.35
Why unable to control HbA1C?	1.54	0.87–2.73	0.13
Are you aware of the clinic services for T2DM people?	0.84	0.48–1.47	0.56
Do you think that the follow-up calls from the clinics are effective regarding your health?	0.54	0.31–0.93	0.02 *
Using metformin	0.68	0.39–1.17	0.16
Using metformin + sulfonylurea	1.29	0.74–2.24	0.35
Using insulin	0.81	0.40–1.63	0.55

uOR: unadjusted odds ratio; CI: confidence interval, *: *p* < 0.05.

**Table 3:** Multivariable binary logistic regression final model for higher resilience (dependent variable).

Independent Variable	B	Wald	Odds Ratio	95% CI	*p*-Value
Moderate–severe Insomnia	−1.55	22.74	0.21	0.11–0.40	<0.001 *
Married	1.20	10.34	3.32	1.60–6.89	0.001 *
Constant	0.84	6.02	2.32		0.01 *

Nagelkerke R^2^ = 0.14. CI: confidence interval, *: *p* < 0.05.

## Data Availability

Data are available from the corresponding author upon resonable request.

## Abbreviations

The following abbreviations are used in this manuscript:

T2DM	Type 2 diabetes mellitus
RHAS	Royal Health Awareness Society
HCC	Healthy Community Clinic
NCDs	non-communicable diseases
HBA1C	glycated hemoglobin (HBA1C)
ISI-A	Insomnia Severity Index—Arabic version
CD-RISC	Connor–Davidson Resilience Scale
OR	Odds ratio
CI	Confidence interval
